# Added Value of One-Month Clinical Data in Predicting Chronic-Stage Motor Function After Ischemic Stroke

**DOI:** 10.3390/jcm15176569

**Published:** 2026-08-26

**Authors:** Yoo Jin Choo, Min Cheol Chang, Ji-Yeon Shin

**Affiliations:** 1 Department of Public Health, Graduate School, Kyungpook National University, Daegu 41566, Republic of Korea; chooyj361@gmail.com; 2 Dr. Chang’s Pain and Rehabilitation Clinic, Daegu 42737, Republic of Korea; wheel633@gmail.com; 3 Pain and Neuromuscular Disorders Research Center, Daegu 42737, Republic of Korea; 4 Department of Preventive Medicine, School of Medicine, Kyungpook National University, Daegu 41944, Republic of Korea

**Keywords:** ischemic stroke, machine learning, predictive modeling, motor recovery, rehabilitation, prognosis

## Abstract

**Background/Objectives**: This study aimed to develop machine learning models—specifically logistic regression (LR), random forest (RF), and deep neural network (DNN) models—using initial and 1-month post-stroke clinical data to predict 6-month upper and lower extremity motor functional outcomes in patients with ischemic stroke. Additionally, we sought to evaluate the potential improvement in discriminative performance and clinical utility achieved by integrating 1-month reassessment data. **Methods**: We analyzed retrospective cohort data from 353 patients with ischemic stroke. Two prediction models were constructed: (1) Model 1, which used only early-stage clinical data, and (2) Model 2, which incorporated both early-stage and 1-month post-stroke clinical data. Model performance and clinical utility were evaluated using the area under the receiver operating characteristic curve (ROC-AUC), DeLong’s test, calibration analysis, decision curve analysis (DCA), and variable importance analysis. **Results**: Although Model 2, which incorporated 1-month data, generally showed an upward trend in discriminative performance across all models for both upper and lower extremity prediction compared to Model 1, a statistically significant improvement was observed only in the LR model for upper extremity prediction (test AUC increased from 0.889 to 0.990; ΔAUC = +0.102, *p* = 0.037). For all other models—including the RF and DNN models for the upper extremity, as well as all lower extremity prediction models—the observed increases in AUC did not reach statistical significance according to DeLong’s test. In calibration analyses, the LR model exhibited the most stable calibration for both extremities. In DCA, Model 2 generally yielded a higher net benefit across most threshold probability ranges compared to Model 1 than Model 1 across most threshold probability ranges. Variable importance analysis indicated a shift in the primary contributing variables from initial motor evoked potential parameters in Model 1 to 1-month clinical functional measures in Model 2. **Conclusions**: Models integrating 1-month reassessment data showed a tendency toward improved discriminative performance compared to those relying solely on initial data. However, as this study was based on a limited sample from a single institution and instability was observed in certain models, external validation using larger, multicenter cohorts is necessary before generalizing these findings.

## 1. Introduction

Ischemic stroke, a major type of stroke caused by vascular occlusion, is associated with high incidence and mortality rates worldwide [1,2]. Survivors often experience chronic sequelae such as motor impairment, which markedly hinders their independence in daily activities and reduces their quality of life [3,4]. Therefore, establishing systematic therapeutic strategies for motor recovery is crucial in the rehabilitation process [5,6], and accurately predicting a patient’s long-term prognosis provides essential information for developing personalized rehabilitation plans [7].

In recent years, machine learning techniques have been actively utilized for predicting disease prognosis in the medical field [8]. Several studies in the field of stroke have attempted to predict functional outcomes in patients with stroke using clinical data from the early stage of onset [9,10,11], but their predictive accuracy has been clearly limited. Functional recovery after ischemic stroke is a dynamic process that unfolds over time; the most pronounced improvements are concentrated within the first month post-onset, and natural recovery largely reaches its limit by 6 months, when patients enter the chronic stage [12,13,14].

Previous studies have largely relied on static data from the early stage of onset, with limited attempts to predict chronic-stage prognosis by incorporating the patient’s condition after this critical recovery period. Furthermore, clinically relevant variables that could enhance predictive precision, such as detailed lesion locations and the frequency of rehabilitation sessions, have rarely been integrated into predictive models. Accordingly, this study aimed to address these limitations. We hypothesized that by additionally incorporating clinical assessments at 1 month post-stroke—a critical period during which the recovery phase is actively progressing—into baseline clinical data, we could more accurately predict motor function at 6 months.

Conventional landmark prediction methods focus on updating the subsequent prognosis using newly acquired information at a specific time point [15]. In contrast, this study framed predictions at stroke onset and at 1 month post-onset not as mutually exclusive alternatives, but as complementary predictive strategies supporting distinct clinical decision-making processes. Specifically, predictions based solely on onset data can be utilized to establish initial rehabilitation plans and counsel patients and their caregivers. Meanwhile, predictions incorporating 1-month follow-up data reflect early recovery trajectories to refine rehabilitation strategies or re-evaluate long-term treatment plans.

Therefore, this study aimed to develop machine learning models, including logistic regression (LR), random forest (RF), and deep neural network (DNN) models, to predict upper and lower extremity motor function at 6 months post-stroke in patients with ischemic stroke, utilizing clinical information from both baseline and 1 month post-onset. Furthermore, by comparing the performance of models using baseline data alone with that of models integrating 1-month data, we sought to quantitatively evaluate the clinical utility and performance enhancement gained from incorporating 1-month reassessment data.

## 2. Materials and Methods

### 2.1. Study Participants

This retrospective cohort study analyzed demographic and clinical data from patients with ischemic stroke treated at Yeungnam University Medical Center, a tertiary care hospital in Daegu, South Korea. The study was approved by the Institutional Review Board of Yeungnam University Medical Center (IRB approval number: YUMC 2023-06-024), and all procedures were performed in accordance with relevant institutional and national guidelines and regulations. Owing to the retrospective nature of this study, the requirement for informed consent was waived by the ethics committee. All eligible patients who visited the hospital between 1 January 2005 and 31 December 2022, and met the predefined inclusion and exclusion criteria were included.

The inclusion criteria were as follows: (1) a diagnosis of ischemic stroke (ICD-10 code I63); (2) completion of transcranial magnetic stimulation (TMS) within 30 days of onset; (3) availability of clinical data, including neurological assessments, collected within 7 days of onset; (4) motor function tests completed at both 1 and 6 months post-onset; (5) evidence of motor function decline post-stroke, defined as a modified Brunnstrom classification (MBC) score < 5 or a Functional Ambulation Category (FAC) score < 4 at baseline; and (6) age ≥ 20 years. The exclusion criteria were as follows: (1) patients with a history of previous stroke; (2) patients whose ischemic stroke was caused by trauma; (3) patients diagnosed with other brain disorders such as tumors or hydrocephalus; and (4) patients with severe cognitive impairments or severe apraxia that could affect motor function prognosis, such as amyotrophic lateral sclerosis, poliomyelitis, dementia, Alzheimer’s disease, or Parkinson’s disease.

A total of 1611 patients were initially identified between 1 January 2005, and 31 December 2022. From this cohort, 1258 patients were excluded due to a history of previous stroke (*n* = 95), hemorrhagic stroke (*n* = 112), traumatic brain injury (*n* = 169), other neurological or brain disorders (*n* = 81), or insufficient clinical data (*n* = 801). The insufficient data category comprised missing National Institutes of Health Stroke Scale (NIHSS) scores (*n* = 759), baseline data (*n* = 13), 1-month follow-up data (*n* = 7), and 6-month follow-up data (*n* = 22). Missing data were handled using complete-case analysis, resulting in a final analytical dataset of 353 patients with no missing values.

**Figure 1:**
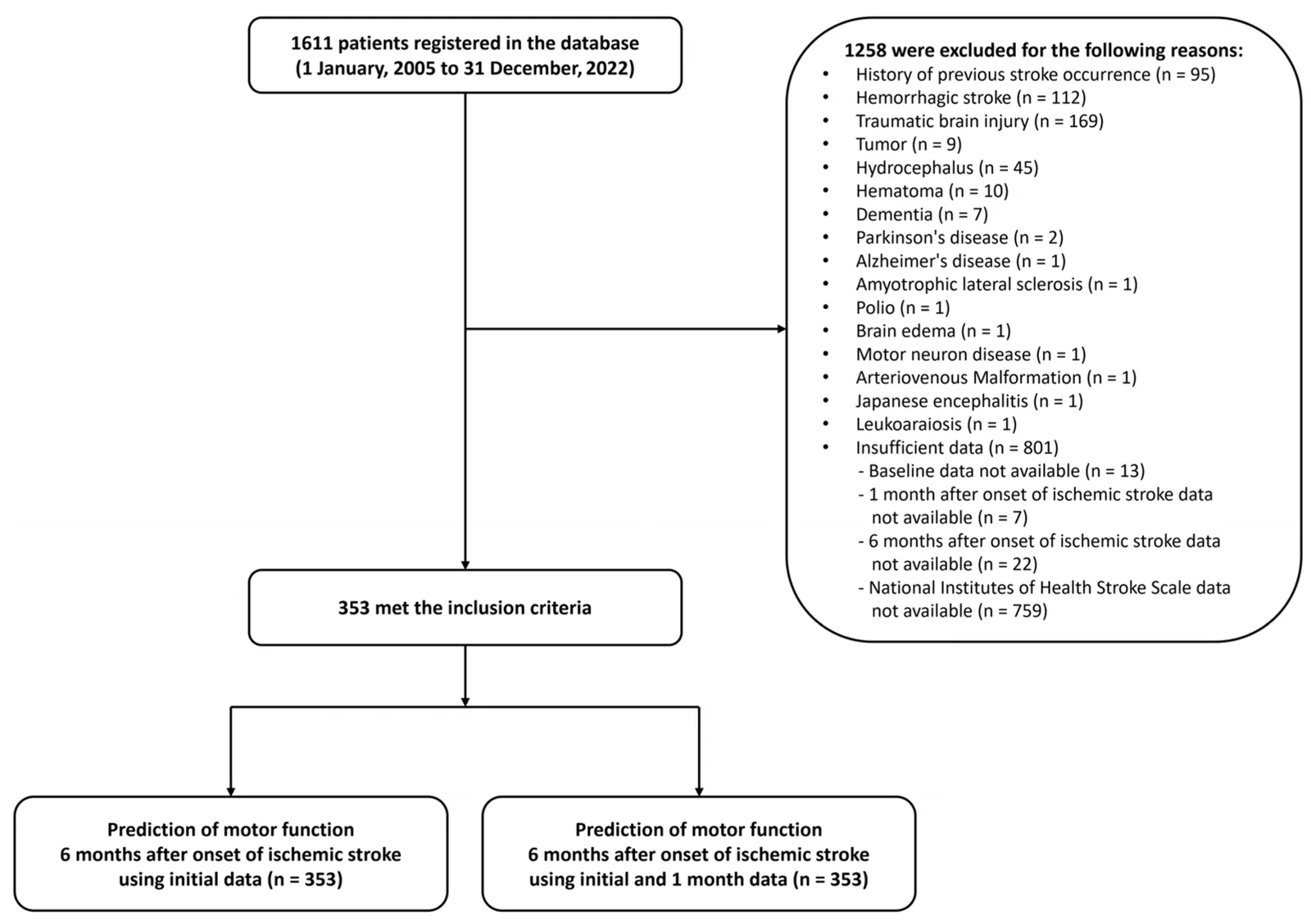
Flow diagram of the patient selection process.

Both Model 1 (using early-stage data exclusively) and Model 2 (additionally incorporating 1-month post-stroke data) were evaluated in the same cohort. Consequently, the datasets for the two models were not independent samples but rather differed solely in the set of clinical variables included. The detailed patient selection process is illustrated in Figure 1.

### 2.2. Data Collection and Variables

#### 2.2.1. Input Variables

We selected a comprehensive set of demographic, clinical, and functional variables based on previous literature indicating their strong predictive value for stroke recovery [9,10,11,16,17]. Input variables were collected at two distinct time points: (1) early-stage assessments, comprising age, sex, ischemic stroke location, medical history, and clinical and functional evaluations measured within 7 days of onset—specifically the NIHSS, modified Rankin Scale (mRS), Medical Research Council (MRC) scale, MBC, FAC, and Korean version of the Mini-Mental State Examination (K-MMSE)—as well as TMS results obtained within 30 ± 7 days of onset; and (2) 1-month post-stroke assessments, comprising MRC, MBC, FAC, and rehabilitation frequency measured at 30 ± 7 days post-stroke. Categorical variables were processed according to their measurement scales: binary variables—including ischemic stroke locations, medical histories, and the presence of motor evoked potential (MEP) responses—were encoded as 0 and 1 (0 indicating absence or a negative finding, and 1 indicating presence or a positive finding), whereas ordinal variables (MRC, MBC, and FAC) were included directly using their original clinical integer grades. A comprehensive list of all input variables used for model training is provided in Supplementary Table S1.

#### 2.2.2. Measurement and Evaluation

All functional assessments were performed by specialized physicians blinded to long-term outcomes.

To assess corticospinal tract integrity, motor evoked potentials (MEPs) were measured from the abductor pollicis brevis (ABP) for the upper extremity and the tibialis anterior (TA) for the lower extremity using a Magstim Novametrix 200 magnetic stimulator (Novametrix, Woburn, MA, USA).

Muscle strength of both upper and lower extremities was evaluated using the MRC scale, ranging from 0 (no muscle contractions present) to 5 (full joint mobility attainable with gravity and strong resistance) [18]. Upper extremity function was evaluated using the MBC, which ranges from 1 (unable to voluntarily move fingers) to 6 (capable of normal movement to grasp or throw a ball; able to button and unbutton a shirt) [19]. Lower extremity function was assessed with the FAC, which ranges from 0 (unable to walk even with assistance from others) to 5 (capable of independent walking in any location) [20].

Neurological deficits were quantified using the NIHSS, which ranges from 0 to 42 (0, no stroke symptoms; 1–4, minor stroke; 5–15, moderate stroke; 16–20, moderate to severe stroke; 21–42, severe stroke) [21]. The degree of disability was graded using the mRS, ranging from 0 (no symptoms) to 6 (dead) [22].

Initial cognitive status, including orientation, memory, attention, and language, was evaluated using the K-MMSE on a 30-point scale, where higher scores indicate better cognitive function [23].

#### 2.2.3. Outcome Variables

The primary outcome variables were the MBC and FAC scores at 6 months post-stroke. For binary classification, these scores were encoded as 0 or 1: an MBC score ≥ 5 and an FAC score ≥ 4 were coded as 1 (favorable outcome), while scores below these thresholds were coded as 0 (poor outcome). These thresholds were selected based on their established clinical relevance: MBC ≥ 5 indicates the ability to perform basic grasp and release functions essential for activities of daily living, and FAC ≥ 4 indicates independent ambulation on level surfaces, both representing meaningful functional independence milestones.

### 2.3. Machine Learning Model Development

We developed machine learning models to predict upper and lower extremity motor function recovery at 6 months post-stroke by constructing Model 1, which utilized only early-stage post-stroke clinical and demographic data, and Model 2, which integrated both early-stage and 1-month clinical assessment data. Models were developed using LR, RF, and DNN algorithms in Python using the scikit-learn library (version 1.2.2). The entire patient dataset was partitioned into training (70%), validation (20%), and test (10%) sets via stratified random sampling to maintain the outcome distribution across the splits. Specifically, for the prediction of upper extremity recovery, this partitioning yielded 148 favorable and 99 poor cases in the training set, 42 favorable and 28 poor cases in the validation set, and 21 favorable and 15 poor cases in the test set. For lower extremity recovery, the event distribution comprised 120 favorable and 127 poor cases for training, 34 favorable and 36 poor cases for validation, and 17 favorable and 19 poor cases for testing. Furthermore, standardization parameters were derived exclusively from the training set and subsequently applied to the validation and test sets. To prevent overfitting and control model complexity, appropriate regularization and constraint techniques were applied to each algorithm, and optimal hyperparameters were selected based on validation set performance (Supplementary Table S2). The test set was used exclusively for final evaluation.

### 2.4. Statistical Analysis

The generalization performance of the machine learning models developed in this study was validated using repeated stratified 5-fold cross-validation (20 repeats), where standardization was applied exclusively within each training fold to prevent data leakage. Overall predictive performance was evaluated using the area under the receiver operating characteristic curve (ROC-AUC). AUC values were interpreted as follows: 0.7–0.79 indicated fair performance, 0.8–0.89 indicated considerable performance, and values of 0.9 or above indicated excellent performance [24]. For the final test-set evaluation, 95% confidence intervals (CIs) for performance metrics including AUC, ΔAUC, sensitivity, specificity, positive predictive value (PPV), negative predictive value (NPV), and Brier score were estimated using 2000 bootstrap resamples; sensitivity, specificity, PPV, and NPV were calculated at a probability threshold of 0.5. The test-set AUCs of Model 1 and Model 2 were compared using DeLong’s test. Furthermore, calibration analysis (Brier score, calibration-in-the-large, and calibration slope) using 1000 bootstrap resamples was performed based on out-of-fold predicted probabilities generated during cross-validation, and clinical utility was evaluated using decision curve analysis (DCA). In addition, to ensure model interpretability, we conducted a variable importance analysis. For LR models, the relative importance of each variable was quantified using adjusted odds ratios [25]. For RF models, importance was determined by the extent to which each variable contributed to reducing Gini impurity at splits across all trees [26]. Due to the high-dimensional feature space and the multilayer structure of DNN models, it is challenging to trace the contribution of individual variables. Therefore, variable importance analysis was not performed for the DNN models [27]. Variable importance analysis was performed solely as a post hoc exploratory step after each model was fully trained, and was used exclusively to aid in the interpretation of the models. The analysis results were not used to influence or modify the input variables, model architectures, or any downstream modeling decisions.

## 3. Results

### 3.1. Characteristics of Study Participants

A total of 353 patients were included in the machine learning model development cohort, with a mean age of 65.1 ± 11.2 years, of whom 208 (58.9%) were male. Regarding lesion locations, the corona radiata was the most frequent, present in 133 patients (37.7%), followed by the basal ganglia in 115 (32.6%), the frontal lobe in 101 (28.6%), and the temporal lobe in 76 (21.5%). Hypertension was the most prevalent underlying comorbidity, affecting 203 patients (57.5%). Baseline assessments indicated moderate functional impairment, with a mean NIHSS score of 9.0 ± 6.1, an mRS score of 3.6 ± 0.9, and a K-MMSE score of 19.0 ± 9.8. On MEP examinations, intact potentials were observed in the ABP in 202 patients (57.2%) and in the TA in 201 (56.9%). Detailed demographic and clinical characteristics are presented in Table 1.

### 3.2. Predictive Performance of Machine Learning Models

**Figure 2:**
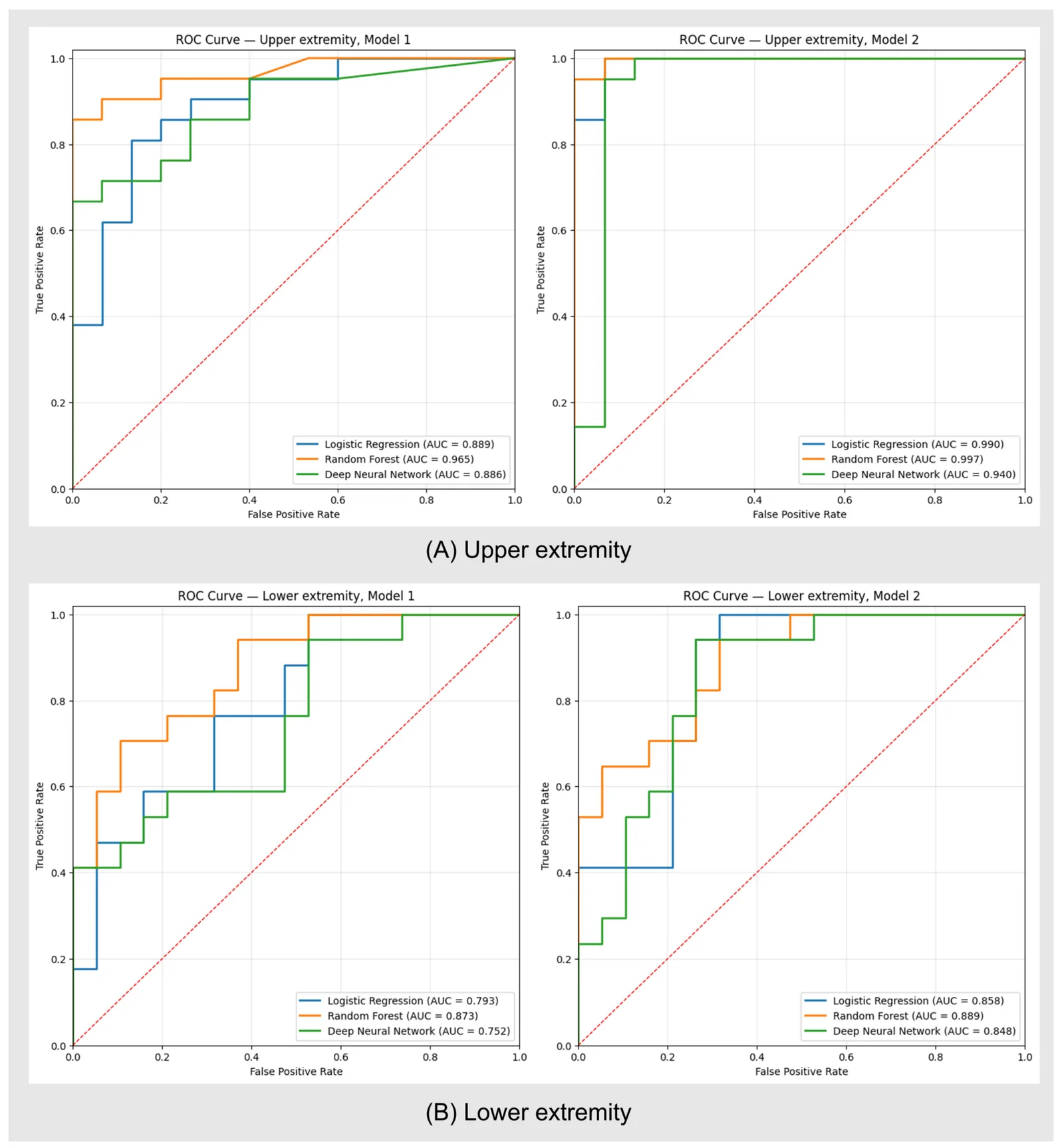
Receiver operating characteristic curves of predictive models for upper and lower extremity recovery. (**A**) Performance for upper extremity functional recovery of Model 1 and Model 2. (**B**) Performance for lower extremity functional recovery of Model 1 and Model 2. Each plot includes three curves representing logistic regression, random forest, and deep neural network models, with their corresponding area under the curve values listed in the legend. The red dashed line represents a random classifier with an AUC of 0.5.

For upper extremity functional recovery prediction, Model 1, which utilized early-stage data, achieved test AUCs of 0.889 (95% CI: 0.765, 0.981) for LR, 0.965 (95% CI: 0.898, 1.000) for RF, and 0.886 (95% CI: 0.764, 0.975) for DNN. In Model 2, which incorporated 1-month clinical assessment data, AUCs improved across all algorithms: 0.990 (95% CI: 0.956, 1.000) for LR, 0.997 (95% CI: 0.981, 1.000) for RF, and 0.940 (95% CI: 0.802, 1.000) for DNN. However, DeLong’s test indicated that this improvement was statistically significant only in the LR model (ΔAUC +0.102 [95% CI: 0.017, 0.208], *p* = 0.037), whereas the differences for RF (*p* = 0.169) and DNN (*p* = 0.412) did not reach statistical significance (Table 2 and Figure 2A).

For lower extremity functional recovery prediction, Model 1 yielded test AUCs of 0.793 (95% CI: 0.631, 0.919) for LR, 0.873 (95% CI: 0.740, 0.966) for RF, and 0.752 (95% CI: 0.571, 0.892) for DNN. Although Model 2 AUCs improved to 0.858 (95% CI: 0.722, 0.966) for LR, 0.889 (95% CI: 0.769, 0.969) for RF, and 0.848 (95% CI: 0.702, 0.960) for DNN, the AUC differences between Model 1 and Model 2 were not statistically significant for any of the algorithms (*p* = 0.227 for LR, *p* = 0.787 for RF, *p* = 0.262 for DNN) (Table 2 and Figure 2B). The corresponding point estimates of the AUCs for individual models in the training and validation sets are provided in Supplementary Table S3.

Regarding classification metrics, the upper extremity LR Model 2 demonstrated relatively well-balanced and favorable classification performance, yielding a sensitivity of 0.905 (95% CI: 0.769, 1.000), a specificity of 0.933 (95% CI: 0.789, 1.000), a PPV of 0.950 (95% CI: 0.833, 1.000), an NPV of 0.875 (95% CI: 0.688, 1.000), and a Brier score of 0.047 (95% CI: 0.014, 0.087). The upper extremity RF Model 2 achieved specificity and PPV values of 1.000 (95% CI: 1.000, 1.000), indicating no false positives. In contrast, despite a high AUC, the upper extremity DNN Model 2 exhibited a collapse in specificity (0.000 [95% CI: 0.000, 0.000]) with a PPV of 0.583 (95% CI: 0.417, 0.723) and an inestimable NPV, reflecting a positive prediction bias that classified all test cases as positive. For lower extremity models, sensitivities across the LR and RF algorithms generally remained lower, ranging from 0.588 to 0.706, indicating a limited ability to detect actual favorable outcomes compared to upper extremity models. Detailed discrimination, classification, and calibration metrics for all models are summarized in Table 3.

### 3.3. Validation Stability and Calibration of Models

In the results of the repeated stratified 5-fold cross-validation (20 repeats, 100 folds in total), LR and RF maintained relatively stable discrimination performance across folds for both Model 1 and Model 2. The largest variation across folds was observed in the 2.5th–97.5th percentile range of [0.733, 0.892] for lower extremity LR Model 1, whereas the smallest variation was observed in [0.956, 0.998] for upper extremity LR Model 2. In contrast, DNN exhibited wider percentile intervals, such as [0.598, 0.853] in lower extremity Model 1 and [0.500, 0.985] in upper extremity Model 2, showing higher variation across folds and lower stability compared to the other two models (Supplementary Table S4).

In the cross-validated out-of-fold analysis, all three algorithms demonstrated lower Brier scores in Model 2 than in Model 1, showing an improvement in overall predictive accuracy. Regarding the calibration-in-the-large metric, LR and RF had 95% CIs containing 0 across all conditions, indicating an absence of overall prediction bias. In contrast, upper extremity DNN yielded 95% CIs below 0 in Model 1 (−0.571, 95% CI: −0.809 to −0.338) and Model 2 (−0.639, 95% CI: −0.819 to −0.452), showing a trend toward systematically producing higher predicted probabilities than actual outcomes. However, no such bias was observed in lower extremity DNN. In the calibration slope analysis, LR exhibited a trend toward underconfidence, with 95% CIs exceeding 1 across all conditions except upper extremity Model 1 (1.143, 95% CI: 0.940 to 1.431). Upper extremity RF yielded calibration slopes of 2.846 (95% CI: 2.358 to 3.505) in Model 1 and 3.471 (95% CI: 2.996 to 4.264) in Model 2, exhibiting a tendency to concentrate predicted probabilities within a narrow range. In contrast, lower extremity RF showed values of 1.351 (95% CI: 1.099 to 1.654) in Model 1 and 1.327 (95% CI: 1.093 to 1.663) in Model 2, demonstrating a less pronounced underconfidence trend compared to the upper extremity. The calibration slope of upper extremity DNN was calculated as 1.564 (95% CI: 1.270 to 2.008) in Model 1 and 3.378 (95% CI: 2.833 to 4.316) in Model 2, displaying underconfidence characteristics, and lower extremity DNN Model 1 also exceeded 1 (1.772, 95% CI: 1.370 to 2.248). In contrast, lower extremity DNN Model 2 yielded 1.035 (95% CI: 0.858 to 1.246), with the 95% CI encompassing 1 and approaching the optimal reference value of 1 (Supplementary Table S5).

The calibration plots for each model are shown in Supplementary Figure S1.

### 3.4. Assessment of Clinical Utility Using Decision Curve Analysis

DCA was used to evaluate the net benefit of each model for 6-month outcome prediction. In the upper extremity, Model 2 demonstrated a higher net benefit compared to Model 1 in the threshold probability range of approximately 0.1–0.9 for LR and approximately 0.3–0.8 for RF. In contrast, in the upper extremity DNN model, the net benefit of Model 2 was higher than that of Model 1 only in the threshold probability range of approximately 0.5–0.8, showing that the ranges of net benefit improvement following the addition of 1-month data differed depending on the algorithm. In the lower extremity, for all three algorithms (LR, RF, and DNN), Model 2 maintained a higher net benefit compared to Model 1 across a wide threshold probability range of approximately 0.2–0.8 (Supplementary Figure S2).

### 3.5. Variable Importance Analysis

To assist in model interpretation, variable importance analysis was performed to evaluate the relative contribution of each variable to the predictions. In upper extremity prediction for Model 1, MEP-related variables demonstrated the highest contributions in both LR and RF (adjusted odds ratio of 2.590 for ABP and Gini importance of 0.318). In Model 2, which integrated 1-month data, both LR and RF highlighted 1-month MBC and MRC scores for muscles including the finger extensor and shoulder abductor as top-contributing variables, demonstrating a shift in the contribution profile from initial MEP-centered indicators toward 1-month clinical recovery indicators (Supplementary Figure S3).

For lower extremity prediction, top-contributing variables differed slightly between algorithms. In LR Model 1, TA, ABP, and FAC scores showed high predictive contributions (adjusted odds ratios of 1.255, 1.187, and 1.118, respectively), whereas in RF Model 1, variable contributions were highest for age, TA, and ABP, in that order (Gini importance of 0.164, 0.100, and 0.076, respectively). In Model 2, both algorithms identified 1-month FAC and MRC scores for muscles such as the hip flexor and knee extensor as top contributors (Supplementary Figure S4).

## 4. Discussion

In this study, machine learning models integrating clinical data from the initial phase and 1 month post-onset were developed to predict the long-term motor function prognosis of patients with ischemic stroke. For both upper and lower extremity function prediction, the model combining initial and 1-month data (Model 2) tended to show an overall improvement in discriminative power compared to the model utilizing only initial data (Model 1). In the prediction of upper extremity function, this improvement reached a statistically significant level in the LR model (ΔAUC = +0.102, *p* = 0.037). In contrast, the RF and DNN models, as well as all lower extremity prediction models, did not reach statistical significance in the DeLong’s test, but a trend toward performance enhancement was consistently observed. This trend was reproduced in the out-of-fold analysis of repeated cross-validation, which may support the potential predictive value of the 1-month reassessment data.

Functional evaluation at 1 month post-stroke may reflect clinical information that can track the course of subsequent motor recovery, which is likely associated with changes in neuroplasticity and motor recovery progressing over several weeks following the initial injury. In the acute and subacute phases, the inhibitory balance between the ipsilesional and contralesional hemispheres changes dynamically, and during this process, the reorganization of the perilesional motor network and the readjustment of interhemispheric mutual inhibition may emerge alongside recovery patterns [28,29]. Therefore, functional assessment at 1 month may serve as an auxiliary indicator reflecting subsequent prognosis better than the initial assessment value.

The variable importance analysis results also showed patterns consistent with this mechanistic interpretation. In Model 1, the variables representing the presence of MEP in the ABP and TA contributed substantially to prediction, which is consistent with previous reports that the presence of MEP in the acute phase is an important indicator for initial prognostic evaluation [30,31]. In contrast, in Model 2 incorporating 1-month data, MBC, FAC, and MRC scores measured at 1 month ranked as the top contributors to prediction. These changes show that the functional status measured after the initial recovery period contributes more strongly to long-term prognostic prediction than baseline neurophysiological indices.

The calibration and clinical utility evaluation results across models demonstrated algorithm-specific characteristics. The LR model showed the most stable calibration among the three algorithms for both upper and lower extremities. This implies that LR calculates predicted probabilities matching actual incidence rates, suggesting that it can be utilized reliably for clinical prognostic prediction in addition to being a highly interpretable linear model. The RF model showed high discriminative power, but tended to concentrate predicted probabilities within a relatively narrow range. This is attributed to the structural characteristic of RF, which averages predictions from multiple decision trees, making predicted probabilities prone to converging toward the middle region rather than extreme values [32], and is consistent with the finding that the calibration slope exceeded the ideal value. The DNN model showed varying patterns depending on the target extremity; the lower extremity DNN Model 2 showed favorable results with a calibration slope of 1.035, approaching ideal calibration, whereas the upper extremity DNN Model 2 exhibited a decrease in specificity and calibration instability despite a high AUC. This performance variation may be related to an increased tendency for overfitting in the high-capacity deep neural network, as a high-dimensional structure containing numerous variables was formed within a limited sample size. In comparison, the fact that LR with a relatively simple structure maintained stable performance suggests that lowering model complexity in limited samples may be advantageous for reducing overfitting and aiding generalization, and future efforts to systematically perform variable selection and dimensionality reduction based on larger-scale data are necessary to address these variations. In the DCA, Model 2 provided higher net benefit than Model 1 across multiple threshold ranges for all three algorithms (LR, RF, and DNN), suggesting that the integration of 1-month data may enhance the clinical utility of prognostic prediction.

While our approach is conceptually aligned with the widely established PREP2 algorithm in stroke upper-limb recovery prediction, it differs in several fundamental aspects. PREP2 is a single-timepoint, rule-based sequential algorithm that predicts 3-month upper extremity function by prioritizing age and shoulder abduction/finger extension strength within days of stroke onset, and sequentially incorporating TMS-elicited MEPs and NIHSS scores for patients with severe initial impairment [33]. Our baseline models are similarly anchored in these established clinical and neurophysiological indicators—including age, corticospinal tract integrity represented by MEPs, and stroke severity represented by the NIHSS—underscoring that early prognosis is grounded in well-validated evidence. Whereas PREP2 focuses on providing a definitive early prognosis at a single acute timepoint, our study investigates how prognostic performance for chronic outcomes is enhanced when initial baseline data are integrated with a 1-month reassessment—a period characterized by active functional recovery. Rather than replacing acute-phase predictive tools, our goal is to complement single-time-point predictions by incorporating a temporal dimension—the recovery trajectory—into prognostic modeling, a longitudinal perspective that established frameworks have not explicitly addressed. Furthermore, methodologically, rather than relying solely on a single rule-based decision tree, we evaluated multiple machine learning algorithms with distinct learning characteristics and expanded the predictive scope beyond the upper extremity to include lower extremity motor outcomes. Thus, while harmonizing with established baseline prediction frameworks, this study offers distinct novelty by demonstrating a complementary, multi-stage prognostic approach that leverages longitudinal reassessment.

Previous studies predicting stroke recovery have primarily relied on single baseline assessments, reporting AUCs ranging from 0.76 to 0.84 [16,17]. However, direct performance comparisons across studies remain limited, as patient populations, predictor sets, outcome definitions, and validation methodologies differ. Moreover, the improved prediction upon incorporating 1-month functional scores is, to some extent, expected, given that these assessments are temporally closer and conceptually related to the 6-month outcomes. Nevertheless, given the scarcity of studies quantitatively evaluating predictive performance by combining baseline and 1-month reassessment data, this study can offer foundational evidence showing that dynamic functional tracking has the potential to complement single-time-point predictions in monitoring recovery trajectories.

This study has several limitations. First, this retrospective study was based on a single tertiary medical center cohort, and selection bias may have occurred because a large majority (approximately 78%) of the total subjects (1611 individuals) were excluded due to reasons such as missing NIHSS scores and loss to follow-up. Because the principal reason for exclusion was the absence of key clinical data, the baseline characteristics of the excluded patients could not be adequately characterized, and systematic differences from the included patients could not be ruled out. In addition, the eligibility requirements for TMS and repeated functional assessments may have yielded a relatively selected rehabilitation population, further limiting generalizability. This limited sample size may have constrained high-dimensional model training, contributing to the instability observed in the upper extremity DNN model and wider cross-validation intervals in some configurations. Second, although regularization, repeated cross-validation, and training-fold-limited standardization were applied to mitigate overfitting and data leakage, these strategies cannot completely replace external validation using high-quality large-scale data, so validation of generalizability is additionally required in future studies. Third, in the TMS evaluation, the presence of MEP was assessed only dichotomously, failing to include continuous quantitative indices such as amplitude, latency, and central motor conduction time. Moreover, additional motor pathways that could affect gait and balance, such as the vestibulospinal and reticulospinal tracts, were not evaluated [34,35,36]. This may partially explain the relatively low lower extremity function prediction performance. Fourth, the rehabilitation therapy variable was defined only as the total number of sessions over 30 days, failing to reflect treatment intensity, time per session, and detailed treatment types, and since rehabilitation frequency is influenced by multifactorial elements such as the patient’s initial severity and medical staff decisions, interpretation requires caution. Fifth, information loss regarding detailed functions may have occurred during the process of dichotomizing ordinal evaluation indicators such as MBC and FAC. In addition, because the 1-month predictors and the 6-month outcomes share the same ordinal scales (MBC and FAC), part of the predictive gain of Model 2 may reflect functional continuity across time points; sensitivity analyses excluding same-scale predictors and models predicting recovery magnitude remain tasks for larger cohorts. Finally, the prolonged 18-year patient enrollment window (2005–2022) introduces potential secular trends due to evolving acute stroke care practices—such as the widespread adoption of endovascular thrombectomy as a standard of care, modifications in thrombolysis protocols, and concurrent changes in rehabilitation strategies—which may have introduced heterogeneity in patient recovery trajectories over time. However, because our strict eligibility criteria limited the final analytical sample to 353 patients, stratifying the dataset chronologically would result in sub-cohort sample sizes and event counts insufficient to ensure stable model estimation. Consequently, temporal validation could not be reliably performed. Future clinical translation will necessitate comprehensive temporal and geographical external validation using large-scale, multi-center datasets.

In conclusion, this study developed machine learning models to predict chronic-stage motor function prognosis by combining initial post-onset data with clinical variables at 1 month, a turning point in recovery. The models incorporating 1-month reassessment data tended to show improved discriminative power compared to the models using only initial data, and this trend was reproduced in repeated cross-validation and led to net benefit improvement in the DCA. This demonstrates the possibility of stepwise prognostic evaluation that reflects the recovery course of patients beyond single-time-point prediction. However, since this study was based on a limited sample from a single institution and instability was observed in some models, external validation through large-scale multicenter cohorts is required for the generalization of the results.

## Figures and Tables

**Table 1:** Characteristics of study participants for machine learning model development.

Characteristics	Value
Number of patients, *n*	353
Male, *n* (%)	208 (58.9)
Mean age, y	65.1 ± 11.2
Days from onset of ischemic stroke to admission, mean ± SD	1.7 ± 3.8
Days from admission to discharge, mean ± SD	42.1 ± 19.1
Lesion of ischemic stroke, *n* (%)	
Frontal lobe	101 (28.6)
Parietal lobe	63 (17.9)
Temporal lobe	76 (21.5)
Occipital lobe	20 (5.7)
Insular	40 (11.3)
Cerebellum	36 (10.2)
Basal ganglia	115 (32.6)
Midbrain	10 (2.8)
Pons	51 (14.5)
Medulla	20 (5.7)
Corona radiata	133 (37.7)
Internal capsule	27 (7.7)
Thalamus	53 (15.0)
Centrum semiovale	9 (2.6)
Medical history, *n* (%)	
Hypertension	203 (57.5)
Diabetes	102 (28.9)
Dyslipidemia	34 (9.6)
Atrial fibrillation	37 (10.5)
Angina pectoris	13 (3.7)
Myocardial infarction	5 (1.4)
Congestive heart failure	4 (1.1)
Arrhythmia	1 (0.3)
Heart failure	1 (0.3)
Valvular heart disease	1 (0.3)
Initial MRC, mean ± SD	
Shoulder abductor	1.8 ± 1.2
Elbow flexor	1.7 ± 1.2
Finger flexor	1.6 ± 1.3
Finger extensor	1.6 ± 1.3
Hip flexor	1.9 ± 1.3
Knee extensor	1.9 ± 1.3
Ankle dorsiflexor	1.7 ± 1.3
Initial MBC, mean ± SD	1.6 ± 1.1
Initial FAC, mean ± SD	0.8 ± 1.1
Initial NIHSS, mean ± SD	9.0 ± 6.1
Initial mRS, mean ± SD	3.6 ± 0.9
Initial K-MMSE, mean ± SD	19.0 ± 9.8
MRC 1 month after onset of ischemic stroke, mean ± SD	
Shoulder abductor	2.6 ± 1.1
Elbow flexor	2.8 ± 1.1
Finger flexor	2.9 ± 1.2
Finger extensor	2.9 ± 1.3
Hip flexor	3.0 ± 1.0
Knee extensor	3.3 ± 0.9
Ankle dorsiflexor	2.9 ± 1.2
MBC 1 month after onset of ischemic stroke, mean ± SD	5.2 ± 1.4
FAC 1 month after onset of ischemic stroke, mean ± SD	2.5 ± 1.0
The presence of MEP, *n* (%)	
Abductor pollicis brevis	202 (57.2)
Tibialis anterior	201 (56.9)
Days from onset of transcranial magnetic stimulation, mean ± SD	17.2 ± 12.1
Number of physical therapy sessions administered within 30 days after the onset of ischemic stroke, mean ± SD	18.4 ± 7.8
Number of occupational therapy sessions administered within 30 days after the onset of ischemic stroke, mean ± SD	15.0 ± 6.9

SD, standard deviation; MRC, Medical Research Council; MBC, modified Brunnstrom classification; FAC, Functional Ambulation Category; NIHSS, National Institutes of Health Stroke Scale; mRS, modified Rankin Scale; K-MMSE, Korean version of the Mini-Mental State Examination; MEP, motor evoked potential.

**Table 2:** Comparison of test-set discrimination performance between early-stage and combined 1-month machine learning models.

	Model 1 ^†^ Test AUC (95% CI)	Model 2 ^‡^ Test AUC (95% CI)	ΔAUC (95% CI)	DeLong *p*
**Upper Extremity**				
Logistic Regression	0.889 (0.765, 0.981)	0.990 (0.956, 1.000)	+0.102 (0.017, 0.208)	0.037
Random Forest	0.965 (0.898, 1.000)	0.997 (0.981, 1.000)	+0.032 (0.000, 0.093)	0.169
Deep Neural Network	0.886 (0.764, 0.975)	0.940 (0.802, 1.000)	+0.054 (−0.084, 0.178)	0.412
**Lower Extremity**				
Logistic Regression	0.793 (0.631, 0.919)	0.858 (0.722, 0.966)	+0.065 (−0.041, 0.181)	0.227
Random Forest	0.873 (0.740, 0.966)	0.889 (0.769, 0.969)	+0.015 (−0.100, 0.138)	0.787
Deep Neural Network	0.752 (0.571, 0.892)	0.848 (0.702, 0.960)	+0.096 (−0.078, 0.272)	0.262

AUC, area under the receiver operating characteristic curve; CI, confidence interval; ΔAUC, difference in AUC between Model 2 and Model 1. ^†^ Model predicting motor function 6 months after ischemic stroke onset using early-stage data. ^‡^ Model predicting motor function 6 months after ischemic stroke by combining early stage and 1 month after ischemic stroke onset data.

**Table 3:** Test-set discrimination, classification, and calibration metrics with 95% confidence intervals.

	AUC	Sensitivity	Specificity	PPV	NPV	Brier
**Upper extremity**						
LR						
Model 1	0.889 (0.765, 0.981)	0.857 (0.692, 1.000)	0.733 (0.500, 0.938)	0.818 (0.632, 0.960)	0.786 (0.555, 1.000)	0.132 (0.067, 0.210)
Model 2	0.990 (0.956, 1.000)	0.905 (0.769, 1.000)	0.933 (0.789, 1.000)	0.950 (0.833, 1.000)	0.875 (0.688, 1.000)	0.047 (0.014, 0.087)
RF						
Model 1	0.965 (0.898, 1.000)	0.952 (0.842, 1.000)	0.733 (0.500, 0.933)	0.833 (0.667, 0.960)	0.917 (0.727, 1.000)	0.141 (0.110, 0.172)
Model 2	0.997 (0.981, 1.000)	0.952 (0.842, 1.000)	1.000 (1.000, 1.000)	1.000 (1.000, 1.000)	0.938 (0.789, 1.000)	0.093 (0.076, 0.113)
DNN						
Model 1	0.886 (0.764, 0.975)	0.952 (0.842, 1.000)	0.533 (0.267, 0.800)	0.741 (0.562, 0.897)	0.889 (0.625, 1.000)	0.176 (0.109, 0.250)
Model 2	0.940 (0.802, 1.000)	1.000 (1.000, 1.000)	0.000 (0.000, 0.000)	0.583 (0.417, 0.723)	NA	0.252 (0.152, 0.360)
**Lower extremity**						
LR						
Model 1	0.793 (0.631, 0.919)	0.588 (0.333, 0.824)	0.737 (0.526, 0.933)	0.667 (0.421, 0.909)	0.667 (0.444, 0.864)	0.189 (0.150, 0.229)
Model 2	0.858 (0.722, 0.966)	0.706 (0.471, 0.913)	0.737 (0.526, 0.933)	0.706 (0.467, 0.913)	0.737 (0.524, 0.929)	0.163 (0.117, 0.210)
RF						
Model 1	0.873 (0.740, 0.966)	0.706 (0.476, 0.929)	0.789 (0.588, 0.950)	0.750 (0.500, 0.941)	0.750 (0.533, 0.941)	0.160 (0.124, 0.198)
Model 2	0.889 (0.769, 0.969)	0.706 (0.478, 0.917)	0.842 (0.667, 1.000)	0.800 (0.571, 1.000)	0.762 (0.565, 0.938)	0.143 (0.096, 0.195)
DNN						
Model 1	0.752 (0.571, 0.892)	0.588 (0.333, 0.812)	0.737 (0.529, 0.933)	0.667 (0.400, 0.900)	0.667 (0.470, 0.864)	0.212 (0.183, 0.243)
Model 2	0.848 (0.702, 0.960)	0.588 (0.350, 0.812)	0.842 (0.650, 1.000)	0.769 (0.500, 1.000)	0.696 (0.500, 0.875)	0.165 (0.114, 0.224)

AUC, area under the receiver operating characteristic curve; PPV, positive predictive value; NPV, negative predictive value. Model 1 uses early-stage data only; Model 2 uses early-stage plus 1-month data.

## Data Availability

The data presented in this study are available from the corresponding author upon reasonable request. The data are not publicly available because they contain sensitive patient information and are subject to Institutional Review Board restrictions.

## Supplementary Materials

The following supporting information can be downloaded at: https://www.mdpi.com/article/10.3390/jcm15176569/s1, Table S1: Input data used in machine learning models; Table S2: Hyperparameters for machine learning models predicting motor function; Table S3: Model discrimination performance across training, validation, and test sets; Table S4: Repeated stratified cross-validation area under the receiver operating characteristic curve of machine learning models; Table S5: Cross-validated out-of-fold calibration performance of machine learning models; Figure S1: Calibration plots of predictive models for upper and lower extremity motor function; Figure S2: Decision curve analysis of predictive models for upper and lower extremity motor function; Figure S3: Variable importance of models predicting 6-month outcomes using early-stage data; Figure S4: Variable importance of models predicting 6-month outcomes using early-stage and 1-month data.

## Abbreviations

The following abbreviations are used in this manuscript:

LR	Logistic regression
RF	Random forest
DNN	Deep neural network
TMS	Transcranial magnetic stimulation
MBC	Modified Brunnstrom classification
FAC	Functional Ambulation Category
NIHSS	National Institutes of Health Stroke Scale
mRS	modified Rankin scale
MRC	Medical Research Council
K-MMSE	Korean version of Mini-Mental State Examination
MEP	Motor evoked potentials
ABP	Abductor pollicis brevis
TA	Tibialis anterior
ROC	Receiver operating characteristic
AUC	Area under the receiver operating characteristic curve
CI	Confidence interval
PPV	Positive predictive value
NPV	Negative predictive value
SD	Standard deviation
