# Supplementary material for: Added Value of One-Month Clinical Data in Predicting Chronic-Stage Motor Function After Ischemic Stroke
Source: J Clin Med. 2026 Aug 26;15(17):6569. doi: 10.3390/jcm15176569 (PMC13566379; doi:10.3390/jcm15176569)
Supplement: Supplementary file 1 [file jcm-15-06569-s001.zip › Supplementary Table S1.pdf]

Supplementary Table S1. Input data used in machine learning models.

**A. Model predicting outcomes six months after onset of ischemic stroke using initial data**			
**Demographic data**			
Age	Sex		
**Clinical data**			
Lesion of stroke	Initial MRC	Initial MBC	
Medical history	Shoulder abductor	Initial FAC	
Initial NIHSS	Elbow flexor	Number of physical therapy	
Initial mRS	Finger flexor	Number of occupational therapy	
Initial K-MMSE	Finger extensor		
The presence of MEP	Hip flexor		
ABP	Knee extensor		
TA	Ankle dorsiflexor		
**B. Model predicting outcomes six months after onset of ischemic stroke using initial and one month data**			
**Demographic data**			
Age	Sex		
**Clinical data**			
Lesion of stroke	Initial MRC	1 month MRC	Initial MBC
Medical history	Shoulder abductor	Shoulder abductor	Initial FAC
Initial NIHSS	Elbow flexor	Elbow flexor	1 month MBC
Initial mRS	Finger flexor	Finger flexor	1 month FAC
Initial K-MMSE	Finger extensor	Finger extensor	Number of physical therapy
The presence of MEP	Hip flexor	Hip flexor	Number of occupational therapy
ABP	Knee extensor	Knee extensor	
TA	Ankle dorsiflexor	Ankle dorsiflexor	

NIHSS, National Institutes of Health Stroke Scale; mRS, modified Rankin scale; K-MMSE, Korean version of mini-mental state classification; MEP, motor evoked potential; ABP, abductor pollicis brevis; TA, tibialis anterior; MRC, Medical Research Council; MBC, modified Brunnstrom classification; FAC, functional ambulation category
