# Supplementary material for: Added Value of One-Month Clinical Data in Predicting Chronic-Stage Motor Function After Ischemic Stroke
Source: J Clin Med. 2026 Aug 26;15(17):6569. doi: 10.3390/jcm15176569 (PMC13566379; doi:10.3390/jcm15176569)
Supplement: Supplementary file 1 [file jcm-15-06569-s001.zip › Supplementary Table S2.pdf]

Supplementary Table S2. Hyperparameters for machine learning models predicting motor function.

	Upper extremity	Lower extremity
**LR**	**Model predicting motor function 6 months after ischemic stroke onset using early-stage data**	
	•Regularization parameter: 0.1<br>•Penalty: l2<br>•Solver: lbfgs<br>•Maximum number of iterations: 100	•Regularization parameter: 0.01<br>•Penalty: l2<br>•Solver: liblinear<br>•Maximum number of iterations: 100
	**Model predicting motor function 6 months after ischemic stroke by combining early-stage and 1 month after ischemic stroke onset data**	
	•Regularization parameter: 0.1<br>•Penalty: l2<br>•Solver: lbfgs<br>•Maximum number of iterations: 100	•Regularization parameter: 0.01<br>•Penalty: l2<br>•Solver: liblinear<br>•Maximum number of iterations: 200
**RF**	**Model predicting motor function 6 months after ischemic stroke onset using early-stage data**	
	•Number of estimators: 300<br>•Maximum depth: 10<br>•Minimum samples required to split an internal node: 2<br>•Minimum samples required at a leaf node: 1<br>•Minimum leaf weight fraction: 0.1<br>•Maximum number of leaf nodes: 100<br>•Out-of-bag score estimate: 84.21%	•Number of estimators: 300<br>•Maximum depth: 10<br>•Minimum samples required to split an internal node: 8<br>•Minimum samples required at a leaf node: 4<br>•Minimum leaf weight fraction: 0.0<br>•Maximum number of leaf nodes: None<br>•Out-of-bag score estimate: 71.66%
	**Model predicting motor function 6 months after ischemic stroke by combining early-stage and 1 month after ischemic stroke onset data**	

	•Number of estimators: 500<br>•Maximum depth: 20<br>•Minimum samples required to split an internal node: 2<br>•Minimum samples required at a leaf node: 1<br>•Minimum leaf weight fraction: 0.0<br>•Maximum number of leaf nodes: 100<br>•Out-of-bag score estimate: 92.31%	•Number of estimators: 500<br>•Maximum depth: 10<br>•Minimum samples required to split an internal node: 10<br>•Minimum samples required at a leaf node: 5<br>•Minimum leaf weight fraction: 0.01<br>•Maximum number of leaf nodes: None<br>•Out-of-bag score estimate: 80.97%
**DNN**	**Model predicting motor function 6 months after ischemic stroke onset using early-stage data**	
	•Hidden Layers: 4 Layers with 256-128-64-32-1 Neurons<br>•Optimizer: SGD<br>•Activation: ReLU<br>•Initial Learning Rate: 0.1<br>•Momentum: 0.8<br>•L2 Penalty Parameter: 0.001<br>•Validation fraction: 0.05<br>•Maximum Number of Iterations: 1000<br>•Number of Iterations with No Change: 10<br>•Batch Size: 64	•Hidden Layers: 2 Layers with 128-64 Neurons<br>•Optimizer: SGD<br>•Activation: ReLU<br>•Initial Learning Rate: 0.01<br>•Momentum: 0.8<br>•L2 Penalty Parameter: 0.4<br>•Validation fraction: 0.1<br>•Maximum Number of Iterations: 3000<br>•Number of Iterations with No Change: 10<br>•Batch Size: 64
	**Model predicting motor function 6 months after ischemic stroke by combining early-stage and 1 month after ischemic stroke onset data**	
	•Hidden Layers: 4 Layers with 256-128-64-32-1 Neurons<br>•Optimizer: SGD<br>•Activation: ReLU<br>•Initial Learning Rate: 1<br>•Momentum: 0.8<br>•L2 Penalty Parameter: 0.001<br>•Validation fraction: 0.05<br>•Maximum Number of Iterations: 1000<br>•Number of Iterations with No Change: 10<br>•Batch Size: 64	•Hidden Layers: 4 Layers with 128-64-32-16 Neurons<br>•Optimizer: SGD<br>•Activation: ReLU<br>•Initial Learning Rate: 0.1<br>•Momentum: 0.7<br>•L2 Penalty Parameter: 0.01<br>•Validation fraction: 0.3<br>•Maximum Number of Iterations: 3000<br>•Number of Iterations with No Change: 15<br>•Batch Size: 64

LR, logistic regression; RF, random forest; DNN, deep neural network
