# Supplementary material for: Added Value of One-Month Clinical Data in Predicting Chronic-Stage Motor Function After Ischemic Stroke
Source: J Clin Med. 2026 Aug 26;15(17):6569. doi: 10.3390/jcm15176569 (PMC13566379; doi:10.3390/jcm15176569)
Supplement: Supplementary file 1 [file jcm-15-06569-s001.zip › Supplementary Table S3.pdf]

Supplementary Table S3. Model discrimination performance across training, validation, and test sets.

	AUC (95% CI)		
	Train	Validation	Test
**Model 1**			
Upper extremity			
LR	0.949	0.981	0.889 (0.765, 0.981)
RF	0.894	0.940	0.965 (0.898, 1.000)
DNN	0.969	0.949	0.886 (0.764, 0.975)
Lower extremity			
LR	0.861	0.847	0.793 (0.631, 0.919)
RF	0.972	0.837	0.873 (0.740, 0.966)
DNN	0.774	0.823	0.752 (0.571, 0.892)
**Model 2**			
Upper extremity			
LR	0.991	0.998	0.990 (0.956, 1.000)
RF	0.974	0.997	0.997 (0.981, 1.000)
DNN	0.936	0.974	0.940 (0.802, 1.000)
Lower extremity			
LR	0.913	0.913	0.858 (0.722, 0.966)
RF	0.967	0.922	0.889 (0.769, 0.969)
DNN	0.947	0.908	0.848 (0.702, 0.960)

LR: logistic regression; RF: random forest; DNN: deep neural network; AUC: area under the receiver operating characteristic curve; CI: confidence interval. Model 1 uses early-stage data only; Model 2 uses early-stage plus 1-month data. The AUCs for the training and validation sets are presented as point estimates for reference without confidence intervals; 95% confidence intervals were calculated exclusively for the test-set AUCs using bootstrapping (2,000 resamples).
