# Supplementary material for: Added Value of One-Month Clinical Data in Predicting Chronic-Stage Motor Function After Ischemic Stroke
Source: J Clin Med. 2026 Aug 26;15(17):6569. doi: 10.3390/jcm15176569 (PMC13566379; doi:10.3390/jcm15176569)
Supplement: Supplementary file 1 [file jcm-15-06569-s001.zip › Supplementary Table S4.pdf]

Supplementary Table S4. Repeated stratified cross-validation area under the receiver operating characteristic curve of machine learning models.

	**Mean AUC for Model 1**	**Mean AUC for Model 2**	**ΔMean AUC**
**Upper extremity**			
Logistic regression	0.921 [0.860, 0.974]	0.981 [0.956, 0.998]	+0.060
Random forest	0.904 [0.842, 0.964]	0.979 [0.951, 0.998]	+0.075
Deep neural network	0.887 [0.779, 0.961]	0.852 [0.500, 0.985]	-0.035
**Lower extremity**			
Logistic regression	0.809 [0.733, 0.892]	0.879 [0.814, 0.935]	+0.070
Random forest	0.821 [0.747, 0.892]	0.890 [0.826, 0.950]	+0.069
Deep neural network	0.745 [0.598, 0.853]	0.867 [0.802, 0.930]	+0.121

AUC, area under the receiver operating characteristic curve. Model 1 uses early-stage data only; Model 2 uses early-stage plus 1-month data. Values represent mean AUC with [2.5th–97.5th percentiles] across 100 folds from repeated stratified 5-fold cross-validation (20 repeats; standardization fitted within training folds). Percentiles indicate empirical spread across folds due to shared training data rather than a formal 95% confidence interval.
