# Supplementary material for: Added Value of One-Month Clinical Data in Predicting Chronic-Stage Motor Function After Ischemic Stroke
Source: J Clin Med. 2026 Aug 26;15(17):6569. doi: 10.3390/jcm15176569 (PMC13566379; doi:10.3390/jcm15176569)
Supplement: Supplementary file 1 [file jcm-15-06569-s001.zip › Supplementary Table S5.pdf]

Supplementary Table S5. Cross-validated out-of-fold calibration performance of machine learning models.

	Brier	Calibration-in-the-large	Calibration slope
**Upper extremity**			
Logistic regression			
Model 1	0.107 (0.088, 0.130)	0.021 (−0.262, 0.283)	1.143 (0.940, 1.431)
Model 2	0.054 (0.041, 0.069)	−0.011 (−0.372, 0.339)	1.293 (1.077, 1.694)
Random forest			
Model 1	0.152 (0.140, 0.165)	0.004 (−0.199, 0.204)	2.846 (2.358, 3.505)
Model 2	0.100 (0.090, 0.109)	0.000 (−0.173, 0.184)	3.471 (2.996, 4.264)
Deep neural network			
Model 1	0.137 (0.119, 0.156)	−0.571 (−0.809, −0.338)	1.564 (1.270, 2.008)
Model 2	0.133 (0.119, 0.149)	−0.639 (−0.819, −0.452)	3.378 (2.833, 4.316)
**Lower extremity**			
Logistic regression			
Model 1	0.184 (0.167, 0.199)	−0.050 (−0.258, 0.173)	1.492 (1.196, 1.878)
Model 2	0.148 (0.130, 0.166)	−0.104 (−0.319, 0.119)	1.516 (1.261, 1.861)
Random forest			
Model 1	0.174 (0.158, 0.191)	−0.005 (−0.219, 0.229)	1.351 (1.099, 1.654)
Model 2	0.136 (0.117, 0.156)	−0.007 (−0.246, 0.235)	1.327 (1.093, 1.663)
Deep neural network			
Model 1	0.206 (0.194, 0.218)	−0.029 (−0.234, 0.186)	1.772 (1.370, 2.248)
Model 2	0.137 (0.115, 0.162)	−0.052 (−0.331, 0.237)	1.035 (0.858, 1.246)

Model 1 uses early-stage data only; Model 2 uses early-stage plus 1-month data. Metrics were computed from out-of-fold predictions of repeated stratified 5-fold cross-validation (20 repeats). For each metric, 95% confidence intervals were obtained by bootstrap resampling of patients (1,000 resamples; 2.5th–97.5th percentiles). A calibration-in-the-large of 0 and a calibration slope of 1 indicate ideal calibration; lower Brier scores indicate better overall accuracy.
